# *Thuniopsis*: A New Orchid Genus and Phylogeny of the Tribe Arethuseae (Orchidaceae)

**DOI:** 10.1371/journal.pone.0132777

**Published:** 2015-08-05

**Authors:** Lin Li, De-Ping Ye, Miao Niu, Hai-Fei Yan, Tie-Long Wen, Shi-Jin Li

**Affiliations:** 1 Key Laboratory of Plant Resources Conservation and Sustainable Utilization, South China Botanical Garden, Chinese Academy of Sciences, Guangzhou, Guangdong, P. R. China; 2 Forest Bureau of Pu’er, Yunnan, P. R. China; 3 University of Chinese Academy of Sciences, Beijing, P. R. China; University of Florida, UNITED STATES

## Abstract

An investigation of a questionable orchid led to the discovery of a new genus and species *Thuniopsis cleistogama*, endemic to Yunnan province, China. It is characterized by having a subglobose corm, a spike-like (racemose) inflorescence, half opened and spurless flowers, a collar-shaped stigma and subglobose capsules. Based on DNA sequence data from three gene regions (nuclear ribosomal ITS, chloroplast *matK* and *trnL*), we investigated its phylogenetic position within the tribe Arethuseae. Phylogenies using maximum likelihood and Bayesian inference support the recognition of *Thuniopsis* as a distinct genus, and suggest its close relationship to the genera *Bletilla*, *Dilochia*, and *Thunia*. The new genus is circumscribed and a description and illustrations of the new species are provided. The phylogenetic relationships among the genera in Arethuseae are accessed. Moreover, our phylogeny also shed light on the phylogenetic positions of several genera which, to date, remain uncertain.

## Introduction

The delimitation of Arethuseae Lindl. (Orchidaceae: Epidendroideae) has historically been difficult and circumscriptions have been variable since the tribe was first described by Lindley [1]. As the current circumscription following Chase *et al*. [2,3], van den Berg *et al*. [4], Pridgeon *et al*. [5] and Freudenstein & Chase [6], it consists of two subtribes: Arethusinae Benth. and Coelogyninae Benth., containing 26 genera and approximately 688 species, which are well represented in eastern Himalayas to southwest China, western Malay Archipelago, Philippines, and New Guinea.

*Thunia* Reichb. f. is a small genus distributed in the Himalayas from India to China and Southeast Asia [5]. Seidenfaden [7] accepted 4 species based on a preliminary study. No further taxonomic revision has been made to *Thunia* since then. It is still uncertain how many species should be recognized. In any case, *Thunia* is generally recognized as a natural group, because its members are similar in morphology and previously treated as the single member of subtribe Thuniinae Schltr. [8]. In the light of the DNA phylogenetic results [4,6,9], all the original members of traditional Coelogyninae and some former members of subtribes Arundininae Dressler, Bletiinae Benth., Glomerinae Schltr. and Thuniinae, including *Aglossorhyncha* Schltr., *Bletilla* Rchb.f., *Dilochia* Lindl., *Glomera* Blume and *Thunia* [8] are now classified in the updated subtribe Coelogyninae, which consists of 21 genera and c. 680 species [2–5]. In contrast to typical members of Coelogyninae *s*.*str*., these five genera possess elongated stems, instead of pseudobulbs. This treatment reflects the complexity of the subtribe with its complicated relationships within and between groups. As Gravendeel & Schuiteman has pointed out in [5]: “with the inclusion of these five genera, Coelogyninae has been transformed from an easily recognized, homogenous subtribe into one that is hard to characterize and even more difficult to delimit from Arethusinae”.

During an exploratory trip to Yunnan province, China, we collected an interesting orchid which displays morphological peculiarities. The plant shows a superficial similarity to *Thunia* in its cylindrical stem covered by distichous, sheathing leaves. Perhaps, based on these characters, it was previously considered as a species of *Thunia* and named as *T*. *cleistogama* [10]. Due to lack of descriptive details and designation of a holotype specimen, such a name is not considered validly published (ICN, Art. 37.1) [11]. Our closer scrutiny revealed that the taxon differs most markedly in having distinctive collar-shaped stigma. In addition, it also shows significant differences in having much smaller, half opened, spurless flowers, a spike-like racemose inflorescence and much smaller, subglobose capsules. The set of its morphological characters did not match *Thunia* or any other closely related genera (see S1 Table).

In this study, the phylogenetic relationships of tribe Arethuseae, especially subtribe Coelogyninae were assessed by using DNA sequence data. The results allowed us to establish the systematic position of this enigmatic species within the tribe more clearly. Our molecular phylogenies using maximum likelihood (ML) and Bayesian inference (BI) clearly identified a novel phylogenetically distinct species, which forms a sister lineage to *Bletilla*, *Dilochia*, and *Thunia*. Because the species cannot be accommodated in any of the known genera based on morphological and molecular data, it is recognized and described here as a new monotypic genus.

## Materials and Methods

### Ethics Statement

The locations of material collected here are neither privately owned lands nor protected areas. The field studies did not involve endangered or protected species included in the Chinese Red Data Book. No specific permits were required for these activities.

### Nomenclature

The electronic version of this article in Portable Document Format (PDF) in a work with an ISSN or ISBN will represent a published work according to the International Code of Nomenclature for algae, fungi, and plants, and hence the new names contained in the electronic publication of a PLOS ONE article are effectively published under that Code from the electronic edition alone, so there is no longer any need to provide printed copies.

In addition, new names contained in this work have been submitted to IPNI, from where they will be made available to the Global Names Index. The IPNI LSIDs can be resolved and the associated information viewed through any standard web browser by appending the LSID contained in this publication to the prefix http://ipni.org/. The online version of this work is archived and available from the following digital repositories: PubMed Central, LOCKSS.

### Morphological observations

Morphological description of *Thuniopsis cleistogama* were based on an examination of fresh and pressed specimens collected from the type locality. In order to investigate its floral morphology in greater detail, mature plants were cultivated in the greenhouse of South China Botanical Garden, Chinese Academy of Sciences (SCBG) for several years. Living and dried specimens of *T*. *cleistogama* for morphological observations were collected in June 2012 from Yunnan (vouchers: *L*. *Li 14*, *18*, *19*). Details of the flowers were examined and photographed under a stereomicroscope (Olympus MD-90). The morphological comparison with other closely related genera was based on study of living plants in the field and in cultivation, herbarium specimens, and information gathered in the literature searches. The specimens examined have been deposited in the herbarium of SCBG (IBSC).

### Taxon sampling

For the tribe-wide analysis, taxa were chosen to represent all genera of Arethuseae [3,5] and most representative variation within larger genera. It was not possible to obtain material of *Aglossorhyncha* (this morphologically uniform genus is generally considered to be closely related to *Glomera*) and *Ischnogyne* Schltr. (this monotypic genus is morphologically similar to *Panisea* (Lindl.) Lindl.). Besides the new taxon, the ingroup sampling included 48 taxa representing 24 of 26 currently recognized genera within the tribe. Two representative species of *Epipactis* Zinn and *Listera* R. Br. were chosen as outgroups based on previous analyses of Arethuseae [4,6,9]. In total, we downloaded 117 sequences for 47 taxa of 25 genera from GenBank. Twenty-one sequences for seven samples were newly generated for this study and have been deposited in the GenBank database. Voucher information and GenBank accession numbers are listed in S2 Table. The ITS and *matK* data matrices and optimal trees were submitted to TreeBASE (submission number 17649, accessible at the URL http://purl.org/phylo/treebase/phylows/study/TB2:S17649) and are also available from the authors upon request.

### DNA extraction, amplification, and sequencing

DNA was extracted from fresh leaves using the modified 2 × CTAB protocol [12]. The analyses presented here used the sequence data from three DNA regions, internal transcribed spacer (ITS) region (ITS1-5.8S-ITS2) of the nuclear ribosomal DNA and plastid DNA (*matK* and *trnL*). These regions have been shown to be valuable in phylogenetic studies within Orchidaceae [4,9,13–18], alone or in combination with each other and/or other DNA regions, where relationships were resolved at a number of taxonomic levels. As far as possible, we used the same DNA samples for these markers. The ITS region was amplified and sequenced with universal primers 17SE and 26SE of Sun *et al*. [19]. For the *matK* region, we used the primers 1R and 3F designed by Ki-Joong Kim and tested by Fazekas *et al*. [20]. For the *trnL* region, PCR amplifications were performed with the primers *trnL*-TAF and *trnL*-TAR developed in this study. The information of primers is listed in S3 Table. All DNA reactions were carried out in a final volume of 25 μL, using 2 μL of DNA extract as template. The amplification mixture contained 12.5 μL Master Mix (Tiangen, Guangzhou, China), 0.5 μL (10 mmol/L) of each primer, 10.5 μL deionized water and 2 μL (15–25 ng/μL) of genomic DNA. The PCR mixtures were performed using a touchdown PCR program [21], consisting of an initial denaturation at 94°C for 3 min, followed by 7 cycles of denaturation at 94°C for 30 s, annealing at 58–52°C for 30 s, and 50 s at 72°C extension. The initial annealing temperature of 58°C was reduced by 1°C after every seven cycles to reach 52°C for the final seven cycles, then, following by 30 cycles of denaturation at 94°C for 30 s, annealing at 52°C for 30 s, and 50 s at 72°C extension and a final extension at 72°C for 8 min. The sequencing reactions were performed by the Invitrogen sequencing service (Invitrogen, Commercial sequencing facility, Guangzhou, China).

### Phylogenetic analyses

Sequence fragments were assembled and edited using Sequencher v.4.5 software package [22]. All the DNA sequences were initially aligned by CLUSTAL X v.2.0 [23] with minor subsequent manual adjustment using the software Se-Al v.2.0a11 [24]. A region of 241bp in the *trnL region* was excluded from the analyses due to ambiguous alignment. Prior to concatenating the individual datasets, congruence of these matrices was evaluated with the partition-homogeneity test [25]. The ILD test was performed in PAUP* v.4.0b10 [26] and implemented using 100 replicates with 10 random addition sequences per replicate and saving ten trees per replicate. ILD tests failed to identify significant conflict between partitions (ITS and *matK*: P = 0.90; ITS and *trnL*: P = 1.00; *matK* and *trnL*: P = 0.98). In addition, we compared the topologies of individual markers to each other to identify the presence of well-supported incongruence and conducted our analysis using four separate datasets: the first with ITS data only; the second with plastid data only; the third with ITS and *matK* combined; the fourth with all data combined. Phylogenetic relationships based on the individual and combined datasets were inferred using unweighted maximum parsimony (MP), Bayesian inference (BI) and maximum likelihood (ML) analyses. BI analyses of individual markers and concatenated datasets were performed in MrBayes v.3.1.2 [27,28]. The best fitting model of evolution was chosen for each marker (ITS, *trnL*, and *matK*) using ModelTest v.3.7 [29]. We chose the models estimated under the Akaike information criterion (AIC) because this method has been shown to perform better than the hierarchical likelihood ratio test when comparing nested models [30]. For each dataset, Markov chain Monte Carlo (MCMC) analyses were performed for 10,000,000 generations, saving one tree each 1000 generations. A conservative burn-in (25%) was applied after checking for stability on the log-likelihood curves and split variances being <0.01. A majority rule consensus tree of the remaining trees was calculated. Branch support was determined by Bayesian Posterior Probabilities (BPP).

ML analyses were implemented using GARLI v.2.0 [31]. The evolutionary models for each marker were specified as described above for the BI analyses. Tree search was conducted using 20,000 generations (genthreshfortopoterm) considering a stochastic algorithm, each resulting in a single best tree. Since no significant differences in the topology were observed when the number of generations was increased, bootstrap support was calculated from 1000 bootstrap replicates [32]. A 50% majority-rule consensus tree was generated using the PAUP* v.4.0d10 [26].

In the maximum parsimony (MP) searches, all characters were treated as unordered, independent, and of equal weight. Gaps were treated as missing data. For each dataset, heuristic searches were conducted with 1000 replicates of random taxon addition, and tree rearrangements using tree bisection-reconnection (TBR) branch swapping, and MULTREES option in effect, and simple addition and ACCTRAN optimization. Bootstrap (BS) analyses were carried out to assess clade support. For all datasets 1000 bootstrap replicates were performed with 100 random sequence addition replicates.

## Results

### Morphological characters

Diagnostically important morphological characters of *Thuniopsis cleistogama* and its related genera, including *Aglossorhyncha*, *Bletilla*, *Dilochia*, *Glomera*, and *Thunia*, are presented in S1 Table.

### Data characteristics of the DNA sequences

Data characteristics and statistics for nrITS, the two plastid regions, and the combined datasets are presented in S4 Table. The number of parsimony-informative characters of the nrDNA ITS sequence (as a whole) is highest (47.24%), followed by the *matK* gene (10.91%). The least variable dataset is *trnL* (intron and exon), with only 4.69% potentially informative sites. As measured by their consistency and retention indices of each dataset, the *matK* and *trnL* perform slightly better than ITS. The much lower CI and RI of the nrITS (0.521 and 0.766) compared with the *matK* sequences (0.821 and 0.851) and *trnL* (0.895 and 0.872) exhibits relatively higher level of homoplasy, which is consistent with the higher rate of the variable sites in this region.

### Maximum parsimony analysis

The strict consensus tree derived from the MP analysis of the plastid region is topologically similar to that based on the nrITS. No strongly supported, incongruent patterns of relationship were detected in the individual analyses. Maximum parsimony heuristic search of the combined ITS and *matK* dataset retrieved 96 most parsimonious trees of 1591 steps (CI = 0.557, RI = 0.769). The placement of *Thuniopsis cleistogama* in the MP analyses is rather unresolved, but it does not group with the species sampled for *Thunia*, instead, species of *Thunia* and *Dilochia* cluster with each other with weak support (MP: 58). Species of *Arundina* and *Anthogonium* nest within the clades of subtribe Coelogyninae with weak support (MP: 54). Beyond that, the strict consensus of these trees is not incongruent with the topologies recovered by both ML and BI analyses of the combined sequence data. Bootstrap support values are generally high in the spine of the tree, but many nodes collapse in the strict consensus tree. The topology provides limited resolution to determine the exact relationships between these members (S1 Fig).

### Bayesian and maximum likelihood analyses

The evolutionary models selected by the Akaike information criteria (AIC) estimator were GTR+I+G for ITS, K81uf+G for *trnL*, and TVM+I+G for *matK*, respectively. Compared with the bootstrap percentage (BP) obtained for maximum parsimony analyses, a much greater number of nodes in the BI tree attained high support (often 1.00 PP). All clades with 50% support in the combined parsimony analysis appeared in BI tree with PP 1.00, and many unresolved clades in the parsimony analysis attained high PP. Bayesian inference analyses recovered more well-supported nodes and received better taxonomic resolution than parsimony-based analyses. In general, there is no significant difference in topologies inferred from Bayesian inference between nrDNA ITS and cpDNA (*matK* and *trnL*) data. Because the trees inferred from plastid sequences do not show any major differences, and are largely unresolved, they are not shown here. The phylogeny of the combined ITS and *matK* follows the ITS tree more closely and is presented in S2 Fig.

**Fig 1:**
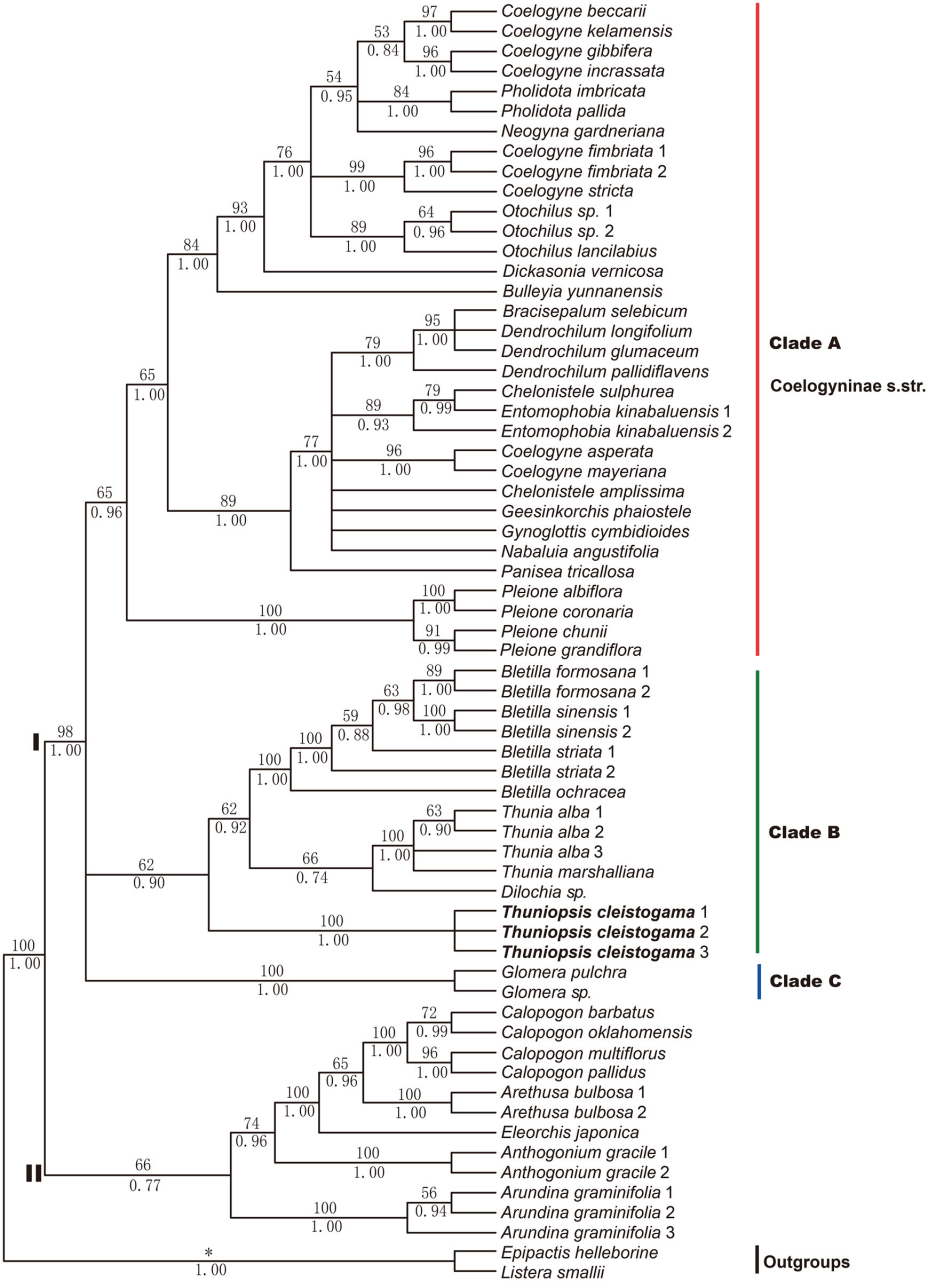
The phylogeny of Arethuseae produced by GARLI and MrBayes using sequence data from nrDNA ITS and cpDNA *matK* datasets. Maximum likelihood bootstrap values and Bayesian posterior probabilities are shown above and below branches, respectively. Bold letters signify clades that are discussed in further detail in the text. The phylogenetic position of *Thuniopsis cleistogama* is highlighted. Asterisk (*) indicate branches resolved only by the BI analysis.

The ML analyses recovered relationships similar to those inferred by BI analyses, differing only in poorly supported internal branches. In some cases, the posterior probabilities are higher than the ML bootstrap percentages, but all clades with high posterior probabilities also receive at least moderate bootstrap support in the ML analyses. The ML phylogeny tree obtained from all three DNA regions by using all ingroup taxa (including those that missing *trnL*) is shown in S3 Fig. The trees generated from all data matrices, both separate and combined, had essentially the same topology. The topology of the combined ITS and *matK* is largely consistent with that recovered in the analysis of the combined dataset of all three DNA regions, but with somewhat higher clade resolution within *Thunia* and its related genera, which was selected to represent the evolutionary relationships within the tribe, with PP from the BI analysis shown where applicable (Fig 1). This analysis included 47 samples that had sequence data from both ITS and *matK* regions, 61 samples that had ITS sequence data, 50 samples that had *matK* sequence data (S2 Table).

The resulting trees indicate two main clades, clade I and clade II. Clade I comprises species currently referred to the subtribe Coelogyninae [2–5], forming a monophyletic group (BI: 1.0, ML: 98), which is resolved as sister to clade II with strong support (BI: 1.0, ML: 100). Clade II including all the sampled species for Arethusinae is weakly or moderately supported as monophyletic (BI: 0.77, ML: 66). Clade I is further divided into clade A, B and C, respectively. Clade A consisting of genera exclusively included in the original interpretation of Coelogyninae *s*.*str*. [8], forms a moderately well-supported monophyletic group (BI: 0.96, ML: 65). Within clade B, the individuals of *Thuniopsis cleistogama* form a monophyletic group (MP: 100, BI: 1.0, ML: 98), weakly supported as sister to the clade comprising *Thunia* and its allies (BI: 0.90, ML: 62). The monophyly of *Bletilla* is well resolved in all of these analyses (MP: 100, BI: 1.0, ML: 100). The sister group relationship of *Thunia* and *Dilochia* is weakly supported in MP (58) and BI analyses (0.74) but moderately well-supported in the ML analysis (66). This clade is weakly recovered as sister to *Bletilla* (BI: 0.92, ML: 62), and then the entire group including *Thunia*, *Dilochia* and *Bletilla* is weakly supported as sister to *T*. *cleistogama* (BI: 0.90, ML: 62). Clade C comprising two sampled species of *Glomera*, is moderately supported as sister to clade B (BI: 0.92) in the BI analyses. Clade B and clade C are resolved as sisters to clade A with high support (BI: 1.0, ML: 98).

All these larger group relationships were resolved in the individual analyses of ITS and the combined dataset (ITS and plastid sequences) by BI and ML analyses, but unresolved in the MP analysis. A considerable improvement was observed in the BI and ML analyses, which indicated that these were able to better accommodate the high levels of homoplasy than an equally weighted maximum parsimony analysis.

## Discussion

### Phylogenetic relationships among the genera within Arethuseae

The ML and BI phylogenies based on two- and three-marker datasets are all largely congruent and support the division of the Arethuseae into two major clades (clade I and II), corresponding to the subtribes Coelogyninae and Arethusinae. The results are in general agreement with previous studies [4,6,9,13]. Moreover, monophyly of the Coelogyninae *s*.*str*. as in Dressler [8], corresponding to the clade A is also clearly present. Our molecular results further indicate the sister relationship of clade A, B and C, which is in accordance with their morphological characteristics. All species of Coelogyninae *s*.*str*. have one or two leaves arising from pseudobulbs; whereas, the remaining species of this subtribe (including clade B and C) are characterized by shared elongated stems, distichous, sheathing leaves, and not forming pseudobulbs. Despite the similarities between clade B and C, *Glomera* differs in basally rhizomatous and branching stems, conduplicate leaves, and a lip without longitudinal ridges. The genus *Aglossorhyncha*, formerly placed in Glomerinae due to its similarity to *Glomera* [3,5,8] has yet to be sequenced, and its placement needs to be clarified. *Glossorhyncha* Ridl., *Ischnocentrum* Schltr., and *Sepalosiphon* Schltr. are generally regarded as synonyms of *Glomera* Blume [3,5].

Some of the traditionally recognized genera, such as *Coelogyne* Lindl., *Dendrochilum* Blume and *Pholidota* Lindl. ex Hook. are found to be not monophyletic. These results are consistent with those recovered in the former study of Gravendeel *et al*. [13]. Phylogenetic analyses indicate that *Coelogyne* species fall into at least two well-supported subclades. Some species nest within species of *Neogyna* Rchb.f. and *Pholidota*; some other species of *Coelogyne* clearly group with species of *Bracisepalum* J.J. Sm., *Chelonistele* Pfitzer, *Dendrochilum*, *Entomophobia* de Vogel, *Geesinkorchis* de Vogel, and *Nabaluia* Ames. The main morphological characters distinguishing these two groups are small or large flower size, persistent or deciduous floral bracts, hairy or glabrous ovaries [13]. As suggested by Chase *et al*. [3], more studies are necessary before substantial changes can be recommended to redefine the circumscription of *Coelogyne* and related genera.

Within clade A, a third major subclade, comprising the sampled species of *Pleione* D. Don, forms a separate early branch sister to the other two major subclades. Morphologically, *Pleione* bears annual pseudobulbs, whereas the others possess long-lived pseudobulbs [5].

In addition, the systematic position of the monotypic genus *Bulleyia* Schltr. was examined for the first time, which represents a sister lineage to the subclade comprising some species of *Coelogyne*, *Dickasonia* L.O. Williams and *Otochilus* Lindl. In addition to caducous floral bracts, *Bulleyia* is identified from its allies by spurred lip and tubular, incurved spur.

As the most basal member of subtribe Arethusinae, *Arundina* Blume is moderately resolved as sister to the other four genera or occupies an isolated position. In contrast to most members in the subtribe, *Arundina* bears elongate reed stems instead of corms. These unusual traits would be consistent with a basal position of the genus. The results agree with the previous finding of van den Berg *et al*. [4]. Freudenstein & Chase [6] provided a mixed result of the position of *Arundina*, being placed in Coelogyninae in the ML method, but nesting within Arethusinae in the MP methods. Chase *et al*. [3] retained its placement in Arethusinae.

### Recognition of a new genus, *Thuniopsis*

Both our BI and ML analyses fail to provide support for inclusion of *Thuniopsis cleistogama* in *Thunia* but clearly support it as an isolated lineage (BI: 1.0, ML: 100), weakly to moderately supported as sister to the clade comprising species of *Bletilla*, *Dilochia* and *Thunia*. Our analyses exhibit a weakly to moderately supported sister group relationship for *Thunia* and *Dilochia* (MP: 58, BI: 0.74, ML: 66). Sister to this clade, we find a strongly supported clade that consists of the sampled species of *Bletilla* (MP: 100, BI: 1.0, ML: 100). The genus *Thunia* as currently recognized could not be retrieved as monophyletic unless *T*. *cleistogama* is exclude. Their relationship clearly needs further investigation, although it appears that *T*. *cleistogama* is not nested in *Thunia*.

The monophyly of *Thunia* is very strongly supported by its high uniformity of vegetative and floral morphology. These terrestrial or lithophytic herbs are characterized by stout, erect and somewhat fleshy stems, distichous, sheathing leaves, and a raceme with characteristically large (40–60 mm in diam.), showy and spurred flowers, ellipsoid or narrowly ellipsoid capsules [5]. It is well represented by *T*. *alba* (Lindl.) Rchb., which is the best known and most widely spread species. *T*. *cleistogama* does not show affinity to *Thunia*, as it has smaller plant, slender and flexible stems (no more than 60 cm long) and strikingly different floral and fruit morphology, such as much smaller, spurless flowers, appearing terminally to form a spike-like racemose inflorescence and much smaller subglobose capsules. The floral bracts are smaller (c. 16 mm long versus 40–50 mm in *T*. *alba*); the sepals are smaller (c. 19 mm long versus 45–60 mm in *T*. *alba*); the petals are smaller (c. 18.5 mm long versus 45–55 mm in *T*. *alba*); the petals are broader than sepals (5.5–6.5 mm/ 3.5–5 mm versus 7–9 mm/ 12–15 mm in *T*. *alba*); the lips are smaller (c. 18 mm long versus 45–50 mm in *T*. *alba*); the rostellum is bilobed at the apex (trilobed in *T*. *alba*); capsules are smaller and rounder (10–13 mm/ 6–9 mm versus 30–35 mm/ 9–10 mm in *T*. *alba*). In addition, the flowers of *T*. *cleistogama* never fully open and the dentate-fimbriate ridges on the disk of the lip are absent. Moreover, all the observed samples of *T*. *cleistogama* in nursery also show high and stable fruit setting rates appearing to be autogamous. However, samples of *T*. *alba* set no fruit under similar condition indicating a conspicuously different pollination mechanism.

Compared with species of *Bletilla* and *Dilochia*, *T*. *cleistogama* can be easily distinguished from both by having persistent floral bracts that are distinctly longer than the ovary. Moreover, unlike most members of Coelogyninae, *T*. *cleistogama* has a subglobose corm, which shows similarity to *Bletilla* and most members of Arethusinae. The unusual morphology may reflect its adaptations to a seasonally dry habitat.

**Fig 2:**
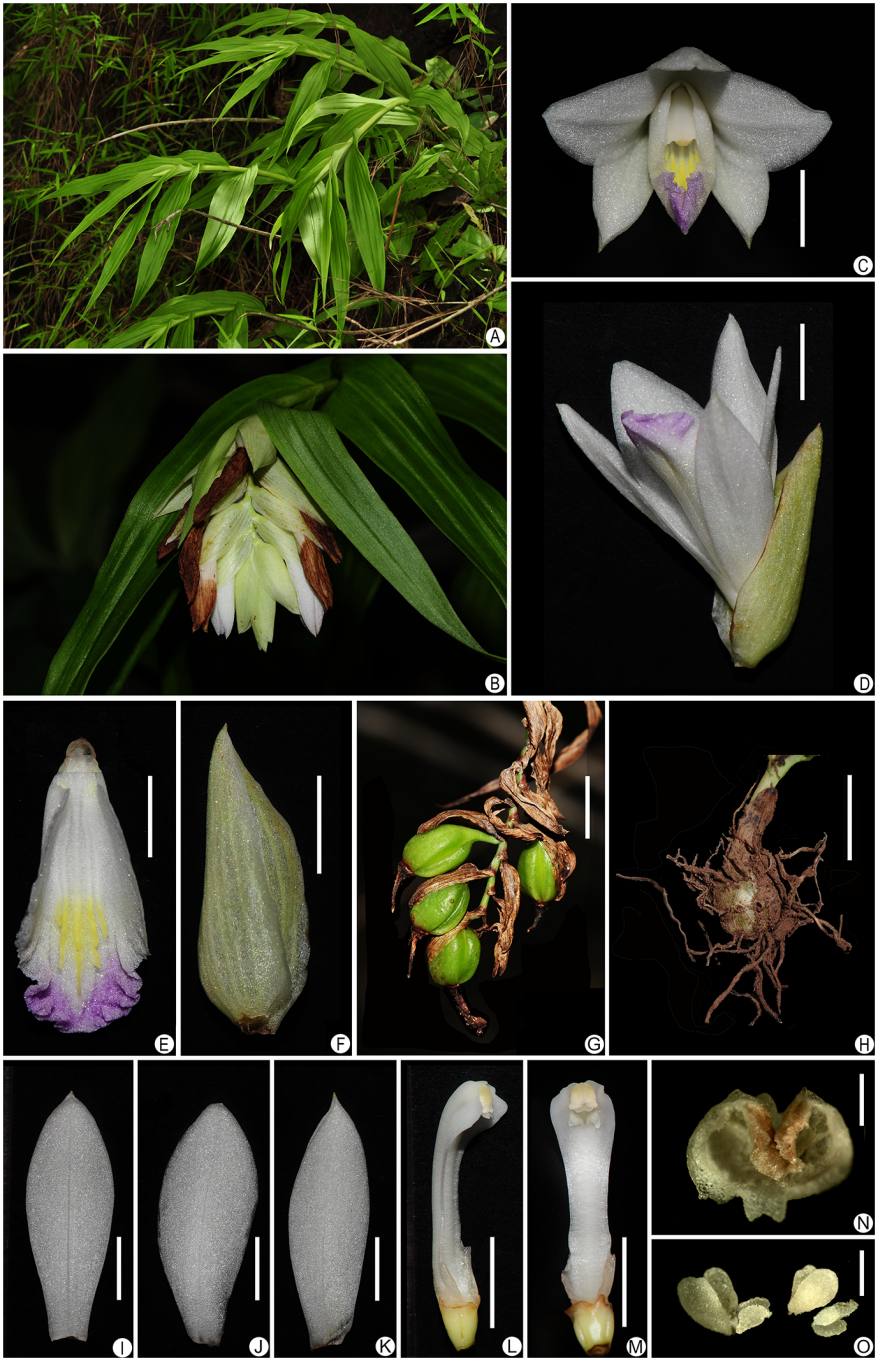
Morphology of *Thuniopsis cleistogama*. A. habit, B. inflorescence, C. flower, frontal view, D. flower, lateral view, E. lip, F. bract, G. fruits, H. corm, I. dorsal sepal, J. petal, K. lateral sepal, L. column, lateral view, M. column, ventral view, N. anther cap, O. pollinaria. Scale bars, 5 mm (C–F; I–M), 10 mm (G), 3 cm (H), 0.5 mm (N–O).

**Fig 3:**
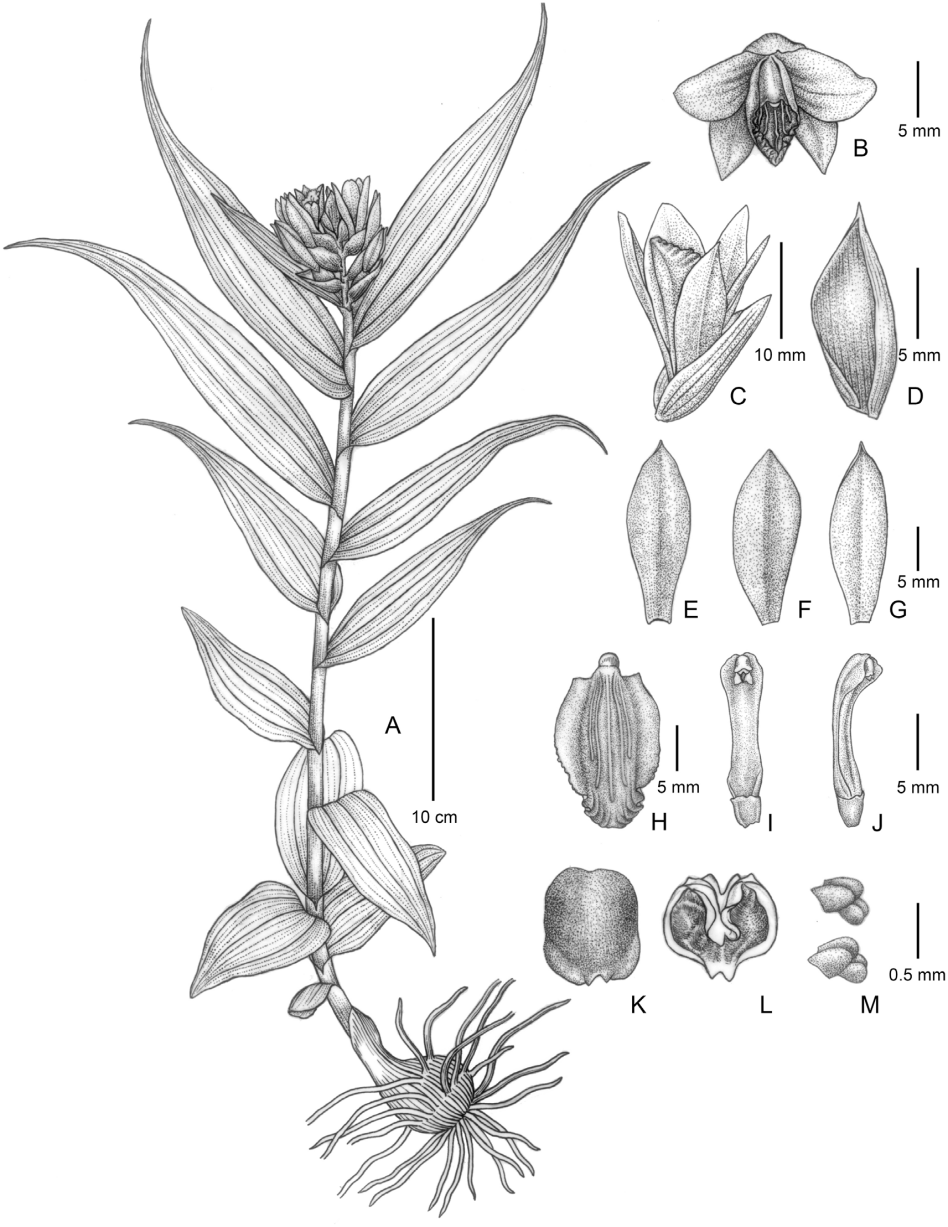
*Thuniopsis cleistogama*. A. a plant in inflorescence, B. flower, front view, C. flower, lateral view, D. bract, E. dorsal sepal, F. petal, G. lateral sepal, H. lip, I. column, ventral view, J. column, lateral view, K. anther cap, dorsal view, L. anther cap, ventral view, M. pollinaria. Drawn by Yun-Xiao Liu from *L*. *Li 19*.

Additionally, the stigma characteristics of *T*. *cleistogama* are found to be distinct when compared to those of related genera (Figs 2 and 3). It has prominent collar-shaped stigma, with a couple of flap-like forward edges folded back, forming a V-shaped opening. So far, this type of stigma is unusual in the subfamily Epidendroideae, and also unique within orchids.

Variation in stigma structure of orchids has been studied by Dannenbaum *et al*. [33] and discussed by Dressler [8], and Freudenstein & Rasmussen [34]. In many taxa of Epidendroideae, the fertile portion of the stigma usually forms a sunken or concave depression, which as Dressler [8] has suggested, appears to be specialized to receive hard pollinia. In tribe Arethuseae, stigma is usually prominent with raised margins, suborbicular, semicircular, or cup-shaped [5]. Our results provide valuable insights into the morphological diversification of stigma in the tribe.

Based on both morphological and molecular evidence, we proposed a new genus *Thuniopsis* to accommodate *T*. *cleistogama*, and describe it below.

## Taxonomic Treatment

### *Thuniopsis* L

#### Li, D. P. Ye & S. J. Li, *gen*. *nov*

[urn:lsid:ipni.org: names: 77147752–1] (Figs 2 and 3). Type: *Thuniopsis cleistogama* L. Li, D. P. Ye & S. J. Li, here designated.

### Diagnosis

Differs from other known genera in having a prominent collar-shaped stigma, with a couple of flap-like top edges, which are folded forming a V-shaped opening on ventral surface of the column. The new genus is also easily recognized by having a subglobose corm, semi-opened and spurless flowers, and subglobose capsules.

### Description

Terrestrial or occasionally lithophytic herbs with subterranean, swollen corms. Corms subglobose to ovoid, 2–3 cm in diam., generally consisting of several internodes, fibrous roots arising from nodes. Stems clustered, cylindrical, slender and flexible, 30–60 cm long, covered by basal scarious scales, sheathing cataphylls below and sheathing leaves above. Leaves 8–14, distichous, alternate, narrowly elliptic-lanceolate to oblong-lanceolate, 10–15 × 1.5–3 cm, often thinly textured, herbaceous or membranous, apex acuminate or long acuminate, with amplexicaul sheaths at base. Inflorescence terminal, borne on young leafy shoots, pendent, synanthous, 6- to 10-flowered, subsessile, racemose, more or less spike-like; floral bracts persistent, membranous, broadly ovate or elliptic, large, longer than ovary, 14–16 mm long, 4.5–5 mm wide, withering soon after anthesis. Flowers alternating in two rows, semi-opening or rarely opening widely, short-lived. Dorsal sepal free, arcuate, oblanceolate or spatulate, 18–19 mm long, 3.5–5 mm wide, mucronate at apex. Lateral sepals similar to dorsal sepal but slightly oblique, 17.5–18.5 mm long, 3.5–5 mm wide. Petals obovate, larger and broader than sepals, 18–18.5 mm long, 5.5–6.5 mm wide, slightly oblique at base, acute or obtuse at apex. Lip basally inserted on the column, cymbiform, oblong-elliptic in outline when flattened, widest at the middle, 17.5–18 mm long, 7.5–11.5 mm wide, entire or obscurely trilobed, spurless, slightly saccate at the base; lateral lobes involute, enclosing column, auriculate, margin inconspicuously erose; middle lobe oblong, apical margin strongly undulate or crisped; disk with five raised lamellate ridges. Column arcuate, clavate, 12–12.5 mm long, dilated and broadly winged at apex, column foot absent. Anthers helmet-shaped, bilocular; pollinia 8, in 2 groups of 4, soft, waxy, unequal in size, with one group distinctly smaller, oblong and laterally compressed, truncate at apex, with granular caudicle, without conspicuous viscidium. Stigma prominent, conspicuously tri-partite, appearing collar-shaped, with a couple of flap-like top edges folded forming a V-shaped opening, upper margin produced into a rostellum, bi-lobed. Flowering in July-September. Capsule subglobose, pendent, ribbed, 10–13mm long, 7–9 mm in dia., enveloped in persistent, scarious floral bracts, with persistent perianths at apex.

### *Thuniopsis cleistogama* L

#### Li, D. P. Ye & S. J. Li, *sp*. *nov*

[urn:lsid:ipni.org:names: 77147753–1] (Figs 2 and 3). Type:—CHINA: Yunnan province, Pu’er, Simao District, ca. 1250 m altitude, 22°54′17"N, 100°43′45"E, 23 Jun 2012, flowered and pressed from plant cultivated in an experimental greenhouse of SCBG, 2 July 2013, *L*. *Li 19* (HOLOTYPE: IBSC!).

— *Thunia cleistogama* D. P. Ye & H. Jiang, The wild orchids in Yunnan: 248, 2010. ***nom*.*nud*.**

### Description

Same as for the genus.

### Etymology

*Thuniopsis* alludes to the resemblance of the plant to some species of *Thunia*. The specific epithet refers to its flowers self-pollinating before opening.

### Distribution, habitat and phenology

Known only from the type locality. This terrestrial orchid prefers shady, moist areas along streams and often grows in weathered soil layer over rock in mixed forests or thickets in dry-hot valleys. The peak flowering period was observed in July and August.

### Conservation status

Endangered, based on the occurrence in an estimated area smaller than 5000 km^2^ and known at fewer than five localities [35].

## Supporting Information

S1 Fig Strict consensus of 96 most parsimonious trees generated from an MP analysis of the combined nrITS and *matK* dataset. Numbers above branches indicate bootstrap values (BS) higher than 50%. Clades that lose resolution in the strict consensus tree are indicated on the figure with an asterisk (*). The phylogenetic position of *Thuniopsis cleistogama* is highlighted.
(PDF)

S2 Fig Comparison of topologies obtained from Bayesian Inference of nrITS (left) and the combined ITS and *matK* dataset (right). Bayesian posterior probabilities are placed above branches. The phylogenetic position of *Thuniopsis cleistogama* is highlighted.
(PDF)

S3 Fig Majority-rule consensus tree obtained by maximum likelihood analyses from a combined dataset of nrITS and cpDNA (*matK* and *trnL*), including those that did not have *trnL* sequence data. Numbers above nodes show bootstrap values. Numbers below nodes indicate Bayesian posterior probabilities recovered by the BI analysis. The Phylogenetic position of *Thuniopsis cleistogama* is highlighted.
(PDF)

S1 Table Diagnostically important morphological characters of *Thuniopsis* and its close relatives. (DOCX)

S2 Table A list of species sampled, vouchers and GenBank accession numbers. The number after the second hyphen in a voucher, e.g., “-1” indicates the individual in a sampled population. Sequences generated in this study are marked with an asterisk (*). Markers noted #### are sequences not available.
(DOCX)

S3 Table Information of primers used in this study. (DOCX)

S4 Table Data characteristics of the DNA sequences. (DOCX)
