# Supplementary figures and images for: Thuniopsis: A New Orchid Genus and Phylogeny of the Tribe Arethuseae (Orchidaceae)
Source: PLoS One. 2015 Aug 5;10(8):e0132777. doi: 10.1371/journal.pone.0132777 (PMC4526666; doi:10.1371/journal.pone.0132777)

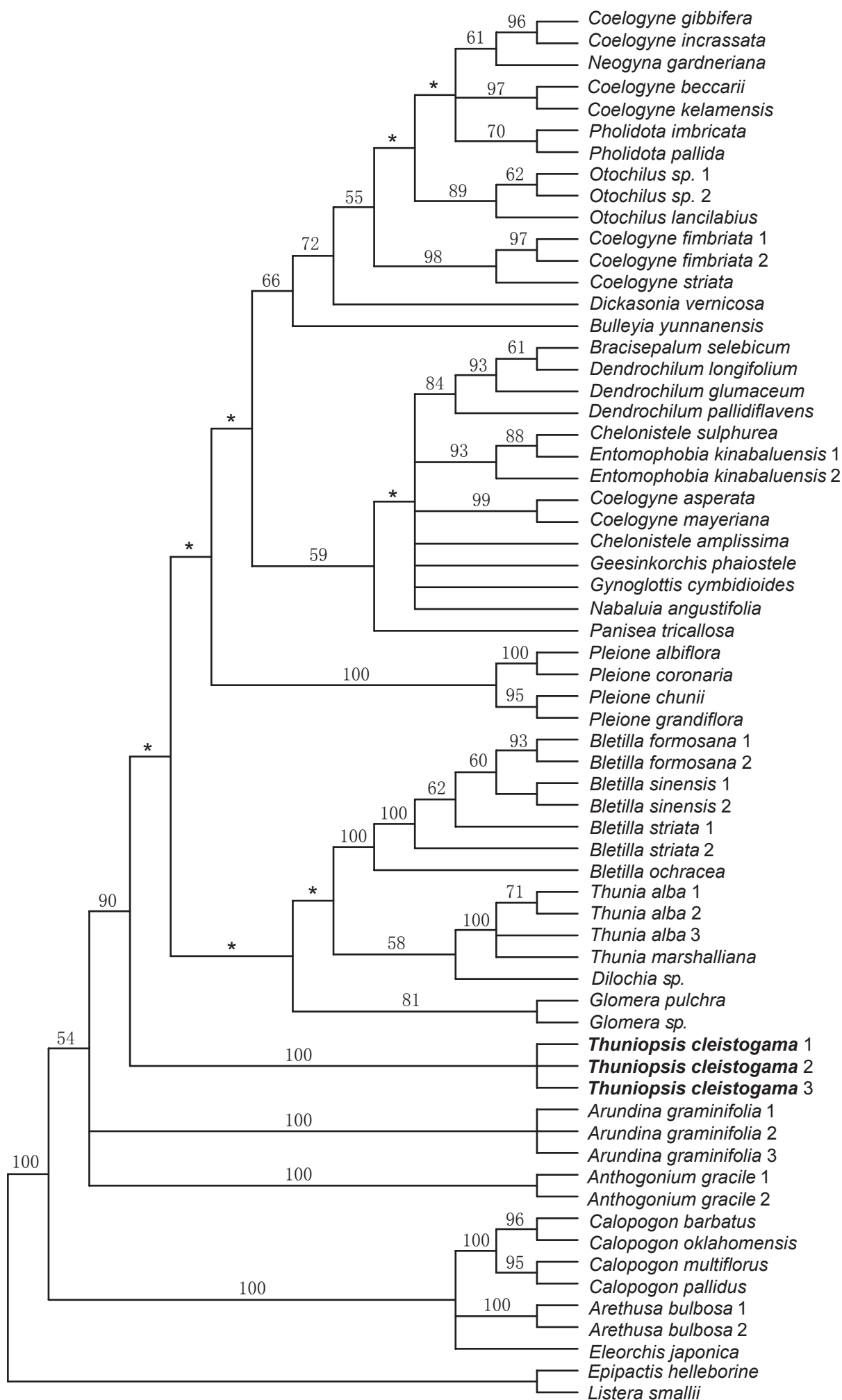

Supplement: S1 Fig — Numbers above branches indicate bootstrap values (BS) higher than 50%. Clades that lose resolution in the strict consensus tree are indicated on the figure with an asterisk (*). The phylogenetic position of Thuniopsis cleistogama is highlighted. (PDF) [file pone.0132777.s001.pdf]

ITS

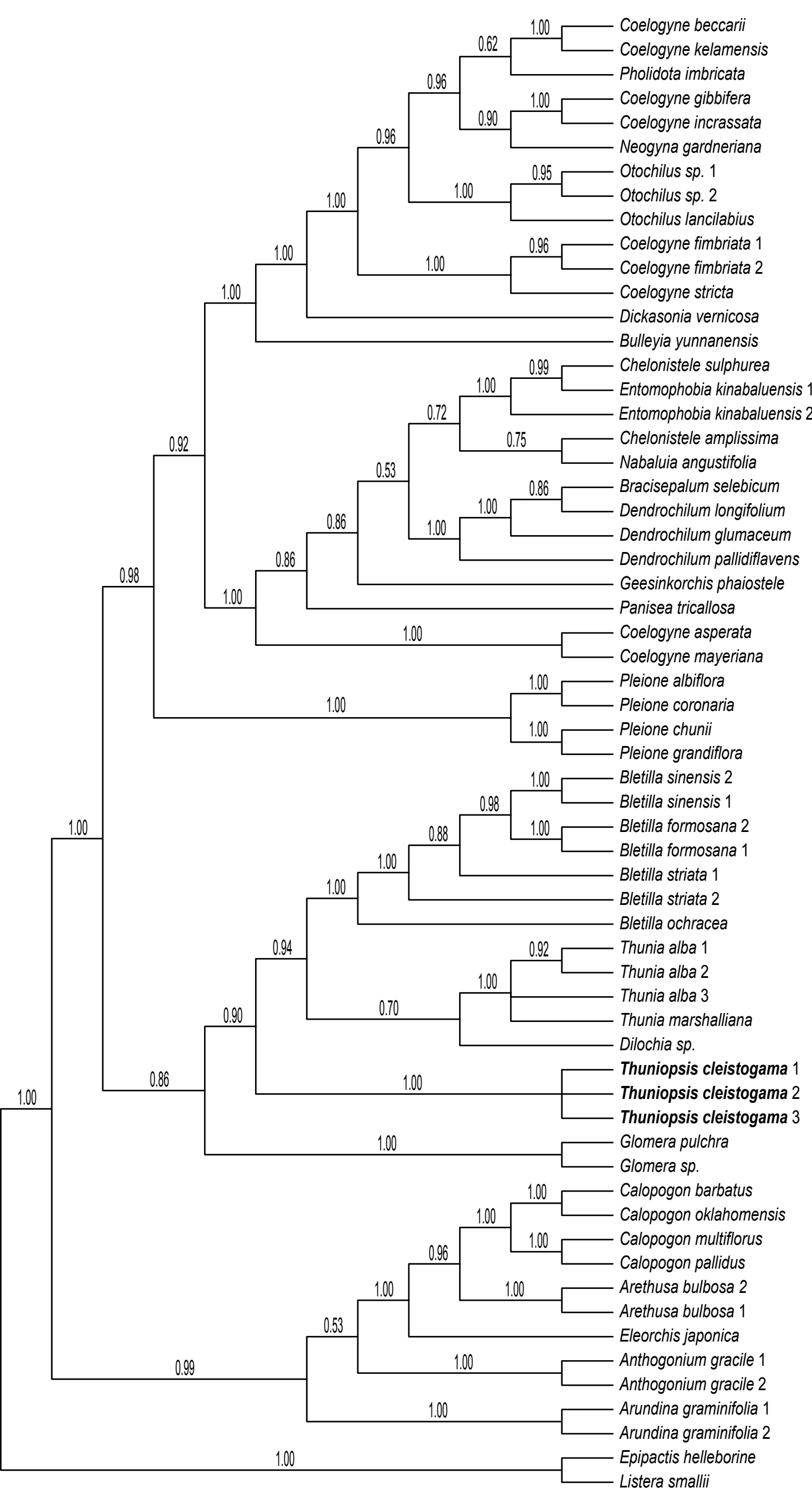

ITS+*matK*

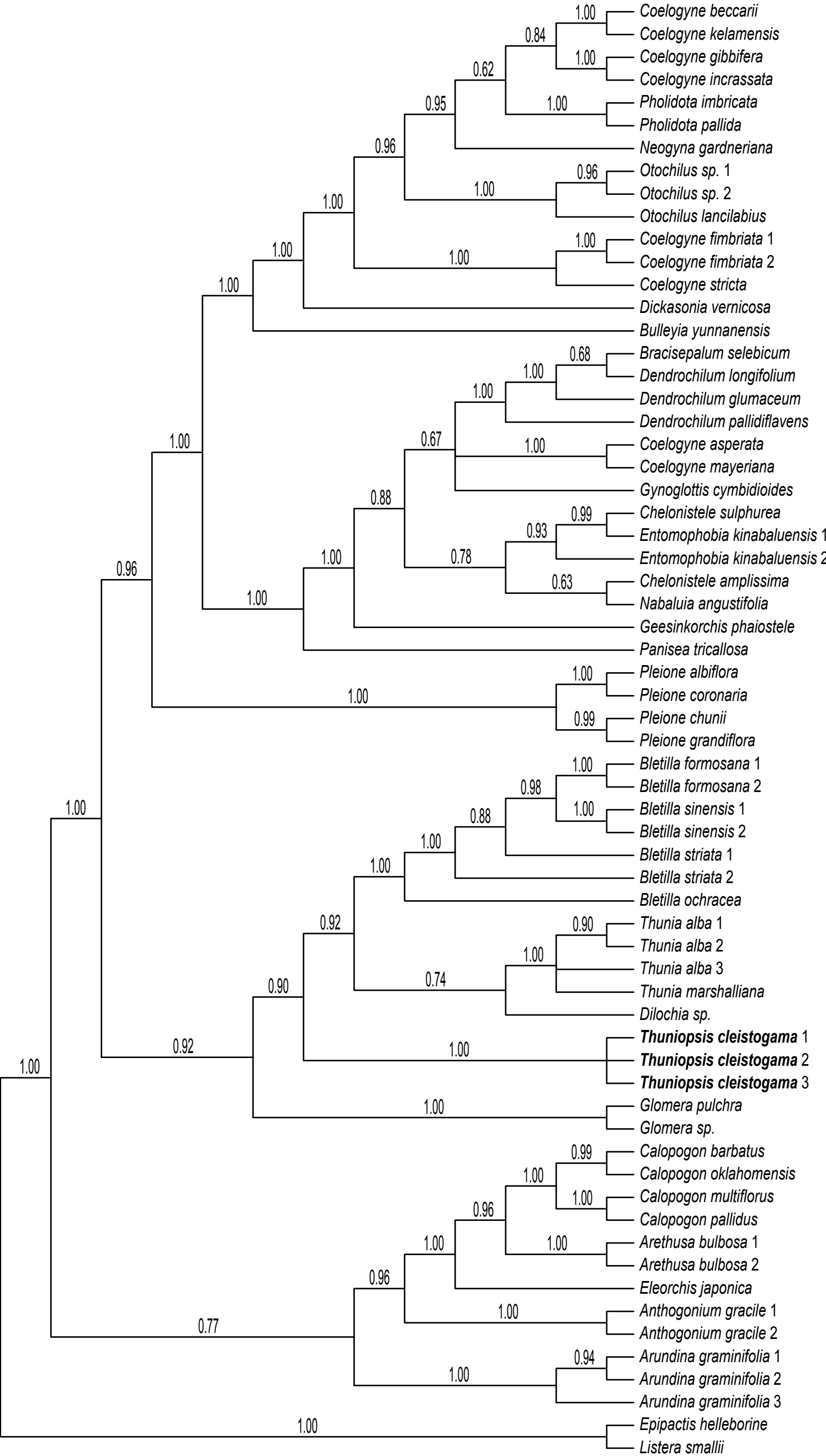

Supplement: S2 Fig — Bayesian posterior probabilities are placed above branches. The phylogenetic position of Thuniopsis cleistogama is highlighted. (PDF) [file pone.0132777.s002.pdf]

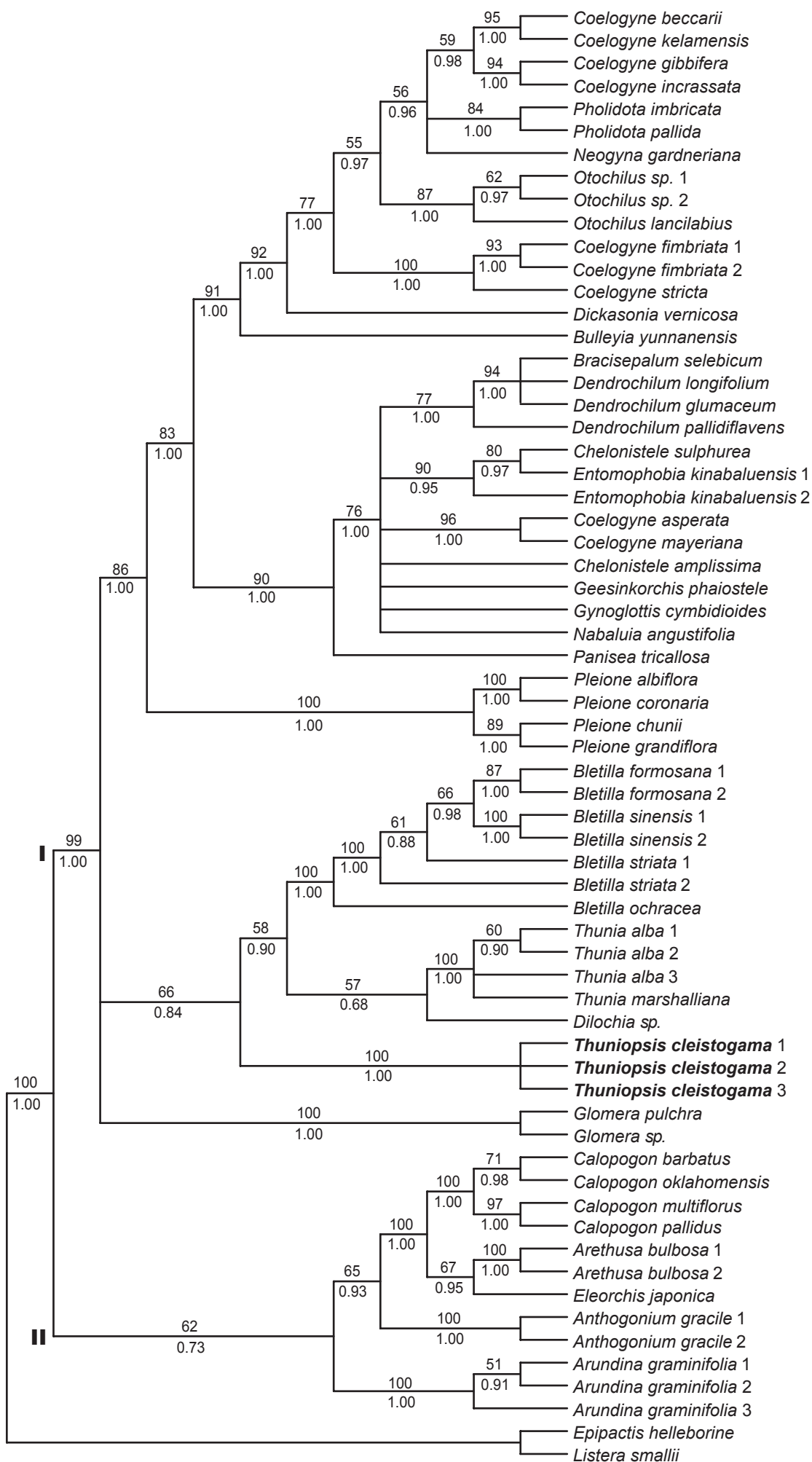

**Clade A**

**Coelogyninae s.str.**

**Clade B**

**Clade C**

**Outgroups**

Supplement: S3 Fig — Numbers above nodes show bootstrap values. Numbers below nodes indicate Bayesian posterior probabilities recovered by the BI analysis. The Phylogenetic position of Thuniopsis cleistogama is highlighted. (PDF) [file pone.0132777.s003.pdf]
