# Supplementary material for: Thuniopsis: A New Orchid Genus and Phylogeny of the Tribe Arethuseae (Orchidaceae)
Source: PLoS One. 2015 Aug 5;10(8):e0132777. doi: 10.1371/journal.pone.0132777 (PMC4526666; doi:10.1371/journal.pone.0132777)
Supplement: S1 Table — (DOCX) [file pone.0132777.s004.docx]

**Table S1. Diagnostically important morphological characters of *Thuniopsis* and its close relatives.**

| Character | *Thuniopsis* | *Aglossorhyncha* | *Bletilla* | *Dilochia* | *Glomera* | *Thunia* |
| --- | --- | --- | --- | --- | --- | --- |
| Corm | Present, subglobose | Absent | Present, irregularly shaped | Absent | Absent | Absent |
| Stem | Slender and flexible, not branching | Basally rhizomatous, often branching, bilaterally flattened | Erect and short, not branching, basal part swelling | Stout, erect, not branching, not fleshy | Basally rhizomatous, often branching | Stout, often erect, not branching, fleshy, basal part swelling |
| Leaf number | Many | Many | 2-4 | Many | Many | Many |
| Leaf vernation | Convolute | Conduplicate | Conduplicate-plicate | Convolute | Conduplicate | Convolute |
| Leaf texture | Herbaceous or membranous | Coriaceous, rarely papyraceous | Papyraceous | Coriaceous | Papyraceous to carnose | Papery or herbaceous |
| Inflorescence | Not branched, spike-like raceme | Not branched, with a single flower or a pair of flowers | Not branched, raceme | Often branched, panicle | Not branched, often dense, subsessile, capitate-like raceme | Not branched, few-flowered, raceme |
| Floral bract | Persistent | Persistent | Deciduous | Deciduous | Persistent | Persistent |
| Flower position | Resupinate | Non-resupinate | Resupinate | Resupinate | Resupinate or not | Resupinate |
| Flower opening | Half opened | Full opened | Full opened | Full opened | Full opened | Full opened |
| Lateral sepal | Free | Free | Free | Free | Often connate | Free |
| Lip hypochile | Spurless, slightly saccate | Spurless, boat-shaped | Spurless | Spurless | Spurred | Spurred |
| Lip mesochile | 5 lamellate ridges | Without ridges | 3 to 5 lamellate ridges | 3 to 5 lamellate ridges | Without ridges | 5 to 9 dentate-fimbriate ridges |
| Column foot | Absent | Absent | Absent | Present | Present or absent | Absent |
| Pollinium number | Eight | Four | Eight | Eight | Four | Four to eight |
| Stigma | Collar-shaped | Suborbicular | Suborbicular to transversely elliptic | Semicircular | Cup-shaped | Semicircular |
| Rostellum | Bilobed | Bilobed | Truncate | Broad | Bilobed | Trilobed |
| Capsule shape | Subglobose | Broadly ellipsoid or ellipsoid | Oblong-fusiform | Broadly ellipsoid | Ellipsoid | Ellipsoid or narrowly ellipsoid |
