# Supplementary material for: Thuniopsis: A New Orchid Genus and Phylogeny of the Tribe Arethuseae (Orchidaceae)
Source: PLoS One. 2015 Aug 5;10(8):e0132777. doi: 10.1371/journal.pone.0132777 (PMC4526666; doi:10.1371/journal.pone.0132777)
Supplement: S2 Table — The number after the second hyphen in a voucher, e.g., “-1” indicates the individual in a sampled population. Sequences generated in this study are marked with an asterisk (*). Markers noted #### are sequences not available. (DOCX) [file pone.0132777.s005.docx]

**Table S2. A list of species sampled, vouchers and GenBank accession numbers.** The number after hyphen in a voucher, e.g., “-1” indicates the individual in a sampled population. Sequences generated in this study are marked with an asterisk (*). Markers noted #### are sequences not available.

| Species Name | Vouchers | ITS | *matK* | *trnL* |
| --- | --- | --- | --- | --- |
| *Anthogonium gracile* Lindl. -1 | Chase O-538 (K) | AF273336 | #### | #### |
| *A. gracile* Lindl. -2 | Chase O-538 (K) | AF521062 | AF263622 | AF519930 |
| *Arethusa bulbosa* L.-1 | D. Goldman 446 (TEX) | AF273339 | #### | #### |
| *A. bulbosa* L.-2 | D. Goldman 446 (TEX) | AF521053 | AF263624 | AF519912 |
| *Arundina graminifolia* (D. Don) Hochr. -1 | Chase O-395 (K) | AF461461 | AF263626 | AF519931 |
| *A. graminifolia* (D. Don) Hochr. -2 | SBB-1053 (DUH) | JN114438 | JN004356 | #### |
| *A. graminifolia* (D. Don) Hochr. -3 | MK Huda 78703(HCU) | #### | KF421845 | #### |
| *Bletilla formosana* (Hayata) Schltr. -1 | AHBZ03 (NJNU) | KF698629 | #### | #### |
| *B. formosana* (Hayata) Schltr. -2 | BFBZ01 (NJNU) | KP866832 | #### | #### |
| *B. sinensis* (Rolfe) Schltr. -1 | BSMZ02 (NJNU) | KP866835 | #### | #### |
| *B. sinensis* (Rolfe) Schltr. -2 | BSMZ03 (NJNU) | KP866836 | #### | #### |
| *B. striata* (Thunb.) Rchb. f. -1 | JJAH02 (NJNU) | KP866825 | #### | #### |
| *B. striata* (Thunb.) Rchb. f. -2 | Chase O-556 (K) | AF461466 | AF263630 | AF519939 |
| **B. ochracea* Schltr*.* | L. Li 06 (IBSC) | KR857328 | KR857335 | KR857342 |
| *Bracisepalum selebicum* J.J. Sm. | Leiden cult. 20446 (L) | AF281120 | AY003873 | #### |
| **Bulleyia yunnanensis* Schltr. | L. Li 21 (IBSC) | KR857329 | KR857336 | KR857343 |
| *Calopogon barbatus* (Walter) Ames | D. Goldman 507 (TEX) | AF273346 | #### | #### |
| *C. multiflorus* Lindl. | D. Goldman 550 (TEX) | AF273342 | #### | #### |
| *C. oklahomensis* D.H. Goldman | D. Goldman 553(TEX) | AF521054 | #### | AF519913 |
| *C. pallidus* Chapm. | D. Goldman 510 (TEX) | AF273353 | #### | #### |
| *Chelonistele amplissima* (Ames & C. Schweinf.) Carr | Leiden cult. 26834 (L) | AF302730 | AF302695 | #### |
| *C. sulphurea* (Blume) Pfitzer | Leiden cult. 21528 (L) | AF302729 | AF302694 | #### |
| *Coelogyne asperata* Lindl. | Leiden cult. 22279(L) | AF281128 | AY003881 | #### |
| *C. beccarii* Rchb. f. | Leiden cult. 32230 (L) | AF302751 | AF302716 | AF463395 |
| *C. fimbriata* Lindl. -1 | Leiden cult. 30759 (L) | AF302745 | AF302710 | #### |
| **C. fimbriata* Lindl. -2 | L. Li 11 (IBSC) | KR857330 | KR857337 | KR857344 |
| *C. gibbifera* J.J. Sm. | SBGO 201 (SING) | AY101966 | AF495864 | AF463385 |
| *C. incrassata* Lindl. | Leiden cult. 932928 (L) | AF463357 | AF463364 | AF463387 |
| *C. kelamensis* J.J. Sm. | Leiden cult. 930568 (L) | AF302750 | AF302715 | AF463388 |
| *C. mayeriana* Rchb.f. | Leiden cult. 30728 (L) | AF281129 | AY003882 | #### |
| *C. stricta* (D. Don) Schltr. | Leiden cult. 30695(L) | AF302757 | AF302722 | #### |
| *Dendrochilum glumaceum* Lindl. | Chase O-624 (K) | AF461465 | AY121730 | AF519933 |
| *D. longifolium* Rchb. f. | Leiden cult. 32110 (L) | AF281121 | AY003874 | #### |
| *D. pallidiflavens* Blume | OYS 041 (UPM) | JF428128 | JF305821 | #### |
| *Dickasonia vernicosa* L.O. Williams | Heidelberg BG 123295 (UGDA) | EF079386 | #### | #### |
| *Dilochia sp.* | Chase O-672 (K) | AF461462 | #### | AF519932 |
| *Eleorchis japonica*  (A.Gray) F.Maek. | D.Goldman 1103(TEX) | AF521055 | AF263657 | AF519914 |
| *Entomophobia kinabaluensis* (Ames) de Vogel -1 | Leiden cult. 970404 (L) | AF461464 | AY121733 | AF519938 |
| *E. kinabuluensis* Rolfe-2 | Leiden cult. 970404 (L) | AF302732 | AF302697 | #### |
| *Epipactis helleborine* (L.) Crantz | Chase O-199 (K) | AF521059 | AF263659 | AF519922 |
| *Geesinkorchis phaiostele* (Ridl.) de Vogel | Leiden cult. 30700 (L) | AF302733 | AF302698 | #### |
| *Glomera pulchra* (Schltr.) J.J. Sm. | Leiden cult. 960835 (L) | AF521079 | #### | AF519963 |
| *Glomera sp.* | Chase O-555 (K) | AF521078 | AY121742 | AF519962 |
| *Gynoglottis cymbidioides* (Rchb. f.) J.J. Sm. | Chase 9452 (K) | #### | FR832773 | #### |
| *Listera smallii* Wiegand | Cameron 1001 (NCU) | AF521058 | AF263668 | AF519920-1 |
| *Nabaluia angustifolia* de Vogel | Leiden cult. 26217 (L) | AF302734 | AF302699 | #### |
| *Neogyna gardneriana* (Lindl.) Rchb. f. | Leiden cult. 970729 (L) | AF302735 | AF302700 | #### |
| *Otochilus lancilabius* Seidenf. | SA 233036 (NHN) | HQ130500 | HQ130494 | #### |
| *O. sp.* -1 | SBB-0587 (DUH) | JN114649 | JN004546 | #### |
| *O. sp.* -2 | SBB-0632 (DUH) | JN114650 | JN004547 | #### |
| *Panisea tricallosa* Rolfe | Leiden cult. 970828 (L) | AF302736 | AF302701 | #### |
| *Pholidota pallida* Lindl. | MK Huda 76820(HCU) | #### | KF421854 | #### |
| *P. imbricata* Hook. | Leiden cult. 21540 (L) | AF302738 | AF302703 | AF463398 |
| *Pleione albiflora* P.J. Cribb & C.Z. Tang | Torelli s.n. (L) | AY101967 | AF503730 | #### |
| *P. chunii* C.L. Tso | van den Berg C290 (K) | AY008471 | AY121732 | AF519936 |
| *P. coronaria* P.J. Cribb & C.Z. Tang | van den Berg C407 (K) | AF461470 | AF503734 | #### |
| *P. grandiflora* (Rolfe) Rolfe | van den Berg C307 (K) | AF461477 | AF503736 | #### |
| *Thunia alba* (Lindl.) Rchb. f. -1 | SBB-0430 (DUH) | JN114721 | JN004618 | #### |
| *T. alba* (Lindl.) Rchb. f. -2 | SBB-0432 (DUH) | JN114723 | JN004620 | #### |
| *T. alba* (Lindl.) Rchb. f. -3 | Chase O-589 (K) | AY008466 | AY121731 | AF519934 |
| **T. marshalliana* Rchb. f. | L. Li 05 (IBSC) | KR857331 | KR857338 | KR857345 |
| ** Thuniopsis cleistogama* L. Li, D.P. Ye & S.J. Li-1 | L. Li 14 (IBSC) | KR857332 | KR857339 | KR857346 |
| ** T. cleistogama* L. Li, D.P. Ye & S.J. Li -2 | L. Li 18 (IBSC) | KR857333 | KR857340 | KR857347 |
| ** T. cleistogama* L. Li, D.P. Ye & S.J. Li -3 | L. Li 19 (IBSC) | KR857334 | KR857341 | KR857348 |
