# Supplementary material for: Thuniopsis: A New Orchid Genus and Phylogeny of the Tribe Arethuseae (Orchidaceae)
Source: PLoS One. 2015 Aug 5;10(8):e0132777. doi: 10.1371/journal.pone.0132777 (PMC4526666; doi:10.1371/journal.pone.0132777)
Supplement: S3 Table — (DOCX) [file pone.0132777.s006.docx]

**Table S3. Information of primers used in this study.**

| Molecular region | Primer name | Primer sequence 5’-3’ |
| --- | --- | --- |
| ITS | 17SE | ACGAATTCATGGTCCGGTGAAGTGTTCG |
|  | 26SE | TAGAATTCCCCGGTTCGCTCGCCGTTAC |
| *matK* | 3F-KIM | CGTACAGTACTTTTGTGTTTACGAG |
|  | 1R-KIM | ACCCAGTCCATCTGGAAATCTTGGTTC |
| *trnL* | trnL-TAF | CGCTACGGACTTGATTGGATTGA |
|  | trnL-TAR | AGAGGGACTTGAACCCTCACGAT |
