# Supplementary material for: Thuniopsis: A New Orchid Genus and Phylogeny of the Tribe Arethuseae (Orchidaceae)
Source: PLoS One. 2015 Aug 5;10(8):e0132777. doi: 10.1371/journal.pone.0132777 (PMC4526666; doi:10.1371/journal.pone.0132777)
Supplement: S4 Table — (DOCX) [file pone.0132777.s007.docx]

**Table S4. Data characteristics of the DNA sequences.**

| DNA region | No. taxa | Aligned length | No. variable characters | No. parsimony-  informative characters | Tree length (steps) | Consistency index (CI) | Retention index (RI) |
| --- | --- | --- | --- | --- | --- | --- | --- |
| ITS region | 61 | 760 | 456(60.00%) | 359(47.24%) | 1367 | 0.521 | 0.766 |
| ITS1 |  | 303 | 203(67.00%) | 168(55.45%) | 661 | 0.502 | 0.755 |
| 5.8S |  | 170 | 43(25.29%) | 20(11.76%) | 64 | 0.719 | 0.858 |
| ITS2 |  | 287 | 210(73.17%) | 171(59.58%) | 611 | 0.547 | 0.791 |
| *matK* gene | 50 | 834 | 162(19.42%) | 91(10.91%) | 218 | 0.821 | 0.851 |
| *trnL* region | 27 | 640 | 78(12.19%) | 30(4.69%) | 95 | 0.895 | 0.872 |
| *trnL* intron |  | 619 | 74(11.95%) | 29(4.68%) | 90 | 0.889 | 0.868 |
| *trnL* 3’ exon |  | 21 | 4(19.05%) | 1(4.76%) | 5 | 1.000 | 1.000 |
| ITS+*matK* | 64 | 1594 | 615(38.58%) | 444(27.85%) | 1591 | 0.557 | 0.769 |
| All plastid data  (except excluded  bases) | 53 | 1474 | 240(16.28%) | 121(8.21%) | 315 | 0.838 | 0.850 |
| All data (except  excluded bases) | 64 | 2234 | 693(31.02%) | 474(21.22%) | 1690 | 0.574 | 0.770 |
